# 2-(3-Methoxy­phen­yl)butane­dinitrile

**DOI:** 10.1107/S1600536809015232

**Published:** 2009-04-30

**Authors:** Wan-Xin Du, Yin Ye, Xian-Wen Wei

**Affiliations:** a College of Chemistry and Materials Science, Anhui Key Laboratory of Functional Molecular Solids, Anhui Normal University, Wuhu 241000, People’s Republic of China

## Abstract

In the title compound, C_11_H_10_N_2_O, the dicyano­ethyl­ene portion has an *anti* conformation. The crystal structure features non-classical C—H⋯N and C—H⋯O inter­actions.

## Related literature

For the synthesis, see: Johnson *et al.* (1962 ▶). The title compound is an inter­mediate in the synthesis of drugs (Obniska *et al.*, 2005 ▶).
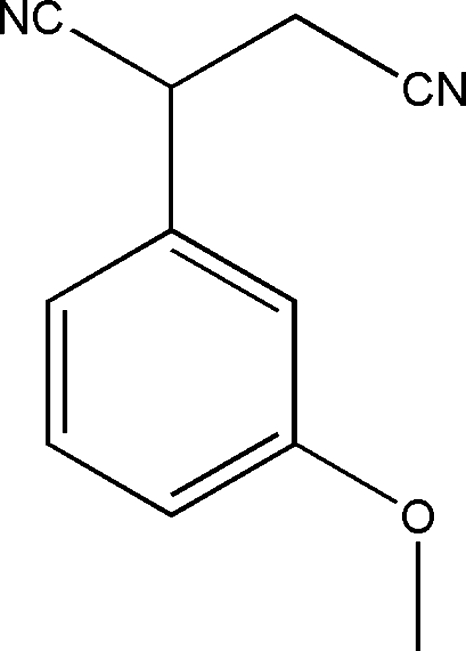


## Experimental

### 

#### Crystal data

C_11_H_10_N_2_O
*M* *_r_* = 186.21
Monoclinic, 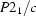

*a* = 5.5263 (8) Å
*b* = 16.105 (2) Å
*c* = 11.0332 (16) Å
β = 97.179 (2)°
*V* = 974.3 (2) Å^3^
*Z* = 4
Mo *K*α radiation
μ = 0.08 mm^−1^
*T* = 298 K
0.4 × 0.2 × 0.1 mm

#### Data collection

Bruker SMART area-detector diffractometer
Absorption correction: none
8042 measured reflections
2210 independent reflections
1963 reflections with *I* > 2σ(*I*)
*R*_int_ = 0.022

#### Refinement

*R*[*F* ^2^ > 2σ(*F* ^2^)] = 0.043
*wR*(*F* ^2^) = 0.128
*S* = 1.04
2210 reflections
128 parameters
H-atom parameters constrained
Δρ_max_ = 0.26 e Å^−3^
Δρ_min_ = −0.22 e Å^−3^

### 

Data collection: *SMART* (Bruker, 2000 ▶); cell refinement: *SAINT* (Bruker, 2000 ▶); data reduction: *SAINT*; program(s) used to solve structure: *SHELXS97* (Sheldrick, 2008 ▶); program(s) used to refine structure: *SHELXL97* (Sheldrick, 2008 ▶); molecular graphics: *SHELXTL* (Sheldrick, 2008 ▶); software used to prepare material for publication: *SHELXTL*.

## Supplementary Material

Crystal structure: contains datablocks I, global. DOI: 10.1107/S1600536809015232/ng2569sup1.cif

Structure factors: contains datablocks I. DOI: 10.1107/S1600536809015232/ng2569Isup2.hkl

Additional supplementary materials:  crystallographic information; 3D view; checkCIF report

## Figures and Tables

**Table 1:** Hydrogen-bond geometry (Å, °)

*D*—H⋯*A*	*D*—H	H⋯*A*	*D*⋯*A*	*D*—H⋯*A*
C8—H8⋯N1^i^	0.98	2.61	3.4864 (17)	150
C10—H10*A*⋯O1^ii^	0.97	2.38	3.2470 (15)	149
C10—H10*B*⋯N2^iii^	0.97	2.60	3.4823 (18)	151

Symmetry codes: (i) 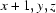; (ii) 
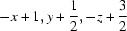
; (iii) 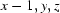.

**Fig. 1.**
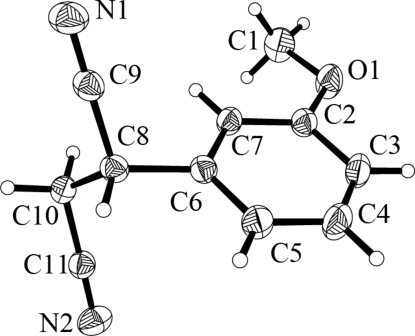
The molecular structure of (I), showing the atom-labelling scheme. Displacement ellipsoids are drawn at the 30% probability level.

**Fig. 2.**
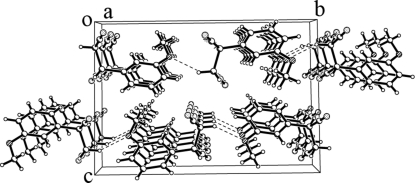
A packing diagram of (I) viewed down the a axis. Dotted lines show the C—H···N and C—H···O hydrogen bonds.

## supplementary crystallographic information

### Comment

The title compound is an important intermediate in drugs synthesis (Obniska *et al.*, 2005). In this paper, we report the structure of the title compound (I). In (I), the succinonitrite moiety adopts an anti conformation. Six atoms of succinonitrite moiety, (N1/C9/C8/N2/C11/C10), almost lie on one plane, the maximum deviations from the mean plane of the succinonitrite being 0.0275 (8) Å. This mean plane is almost perpendicular to the phenyl mean plane with a dihedral angle of 87.55 (6) Å. The crystal packing is stabilized by two intermolecular non-classic C—H···N hydrogen bonds and one intermolecular non-classic C—H···O hydrogen bond.

### Experimental

The compound (I) was obtained by reaction of (*Z*)-ethyl-2-cyano-3-(4-methoxyphenyl)acrylate and NaCN in ethanol-water mixture according to the reported method (Johnson *et al.*, 1962). Single crystals suitable for X-ray diffraction were obtained by evaporation of an ethanol solution at room temperature.

### Refinement

All non-hydrogen atoms were refined anisotropically. H atoms bonded to C atoms were introduced at calculated positions and refined using a riding model with C—H distances of 0.93–0.97 Å. In all cases, the H-atom *U*_iso_(H) is 1.2 times *U*_eq_ of the parent atom.

### Crystal data

**Table:**

C_11_H_10_N_2_O	*F*(000) = 392
*M**_r_* = 186.21	*D*_x_ = 1.270 Mg m^−^^3^
Monoclinic, *P*2_1_/*c*	Mo *K*α radiation, λ = 0.71073 Å
Hall symbol: -P 2ybc	Cell parameters from 5752 reflections
*a* = 5.5263 (8) Å	θ = 2.3–27.4°
*b* = 16.105 (2) Å	µ = 0.08 mm^−^^1^
*c* = 11.0332 (16) Å	*T* = 298 K
β = 97.179 (2)°	Block, colorless
*V* = 974.3 (2) Å^3^	0.4 × 0.2 × 0.1 mm
*Z* = 4	

### Data collection

**Table:**

Bruker SMART area-detector diffractometer	1963 reflections with *I* > 2σ(*I*)
Radiation source: fine-focus sealed tube	*R*_int_ = 0.022
graphite	θ_max_ = 27.5°, θ_min_ = 2.3°
φ and ω scans	*h* = −7→7
8042 measured reflections	*k* = −20→20
2210 independent reflections	*l* = −14→14

### Refinement

**Table:**

Refinement on *F*^2^	Primary atom site location: structure-invariant direct methods
Least-squares matrix: full	Secondary atom site location: difference Fourier map
*R*[*F*^2^ > 2σ(*F*^2^)] = 0.043	Hydrogen site location: inferred from neighbouring sites
*wR*(*F*^2^) = 0.128	H-atom parameters constrained
*S* = 1.04	*w* = 1/[σ^2^(*F*_o_^2^) + (0.0729*P*)^2^ + 0.1612*P*] where *P* = (*F*_o_^2^ + 2*F*_c_^2^)/3
2210 reflections	(Δ/σ)_max_ = 0.001
128 parameters	Δρ_max_ = 0.26 e Å^−^^3^
0 restraints	Δρ_min_ = −0.21 e Å^−^^3^

### Special details

**Table:**

Geometry. All e.s.d.'s (except the e.s.d. in the dihedral angle between two l.s. planes) are estimated using the full covariance matrix. The cell e.s.d.'s are taken into account individually in the estimation of e.s.d.'s in distances, angles and torsion angles; correlations between e.s.d.'s in cell parameters are only used when they are defined by crystal symmetry. An approximate (isotropic) treatment of cell e.s.d.'s is used for estimating e.s.d.'s involving l.s. planes.
Refinement. Refinement of *F*^2^ against ALL reflections. The weighted *R*-factor *wR* and goodness of fit *S* are based on *F*^2^, conventional *R*-factors *R* are based on *F*, with *F* set to zero for negative *F*^2^. The threshold expression of *F*^2^ > σ(*F*^2^) is used only for calculating *R*-factors(gt) *etc*. and is not relevant to the choice of reflections for refinement. *R*-factors based on *F*^2^ are statistically about twice as large as those based on *F*, and *R*- factors based on ALL data will be even larger.

### Fractional atomic coordinates and isotropic or equivalent isotropic displacement parameters (Å^2^)

**Table:**

	*x*	*y*	*z*	*U*_iso_*/*U*_eq_	
C6	0.6739 (2)	0.85144 (7)	0.67253 (10)	0.0379 (3)	
C7	0.5211 (2)	0.80066 (7)	0.73097 (11)	0.0406 (3)	
H7	0.4177	0.8237	0.7822	0.049*	
C5	0.8292 (3)	0.81733 (9)	0.59747 (12)	0.0518 (3)	
H5	0.9321	0.8512	0.5588	0.062*	
C2	0.5237 (2)	0.71565 (7)	0.71258 (11)	0.0439 (3)	
C3	0.6789 (3)	0.68150 (8)	0.63618 (14)	0.0551 (4)	
H3	0.6799	0.6245	0.6231	0.066*	
C4	0.8306 (3)	0.73211 (9)	0.58018 (15)	0.0622 (4)	
H4	0.9358	0.7090	0.5299	0.075*	
C8	0.6709 (2)	0.94492 (7)	0.69077 (10)	0.0384 (3)	
H8	0.7891	0.9698	0.6423	0.046*	
C10	0.7385 (2)	0.97151 (7)	0.82538 (11)	0.0414 (3)	
H10A	0.7311	1.0315	0.8311	0.050*	
H10B	0.6209	0.9485	0.8744	0.050*	
C9	0.4279 (2)	0.97888 (7)	0.64751 (11)	0.0435 (3)	
O1	0.37911 (19)	0.66090 (5)	0.76429 (10)	0.0605 (3)	
C1	0.2421 (3)	0.69035 (10)	0.85616 (15)	0.0638 (4)	
H1A	0.3505	0.7141	0.9218	0.096*	
H1B	0.1545	0.6450	0.8867	0.096*	
H1C	0.1286	0.7318	0.8221	0.096*	
N1	0.2393 (2)	1.00418 (8)	0.61421 (12)	0.0604 (3)	
C11	0.9826 (2)	0.94328 (7)	0.87350 (10)	0.0415 (3)	
N2	1.1722 (2)	0.92210 (8)	0.91331 (11)	0.0581 (3)	

### Atomic displacement parameters (Å^2^)

**Table:**

	*U*^11^	*U*^22^	*U*^33^	*U*^12^	*U*^13^	*U*^23^
C6	0.0380 (6)	0.0381 (6)	0.0376 (5)	−0.0021 (4)	0.0054 (4)	−0.0030 (4)
C7	0.0415 (6)	0.0363 (6)	0.0462 (6)	−0.0003 (4)	0.0139 (5)	−0.0042 (4)
C5	0.0540 (8)	0.0522 (7)	0.0537 (7)	−0.0070 (6)	0.0239 (6)	−0.0074 (5)
C2	0.0443 (6)	0.0368 (6)	0.0518 (6)	−0.0028 (5)	0.0112 (5)	−0.0029 (5)
C3	0.0617 (8)	0.0384 (6)	0.0679 (8)	0.0001 (5)	0.0188 (6)	−0.0143 (6)
C4	0.0668 (9)	0.0565 (8)	0.0696 (9)	−0.0002 (7)	0.0339 (7)	−0.0175 (7)
C8	0.0364 (6)	0.0373 (6)	0.0417 (6)	−0.0028 (4)	0.0053 (4)	0.0024 (4)
C10	0.0399 (6)	0.0366 (5)	0.0467 (6)	0.0036 (4)	0.0019 (5)	−0.0048 (4)
C9	0.0436 (7)	0.0397 (6)	0.0460 (6)	−0.0049 (5)	0.0007 (5)	0.0055 (5)
O1	0.0694 (7)	0.0352 (5)	0.0829 (7)	−0.0077 (4)	0.0339 (5)	−0.0025 (4)
C1	0.0727 (10)	0.0539 (8)	0.0708 (9)	−0.0093 (7)	0.0331 (8)	0.0033 (7)
N1	0.0479 (7)	0.0619 (7)	0.0679 (8)	0.0001 (5)	−0.0065 (5)	0.0126 (6)
C11	0.0429 (6)	0.0402 (6)	0.0413 (6)	0.0009 (5)	0.0050 (5)	−0.0025 (4)
N2	0.0469 (6)	0.0739 (8)	0.0525 (6)	0.0129 (5)	0.0023 (5)	−0.0037 (5)

### Geometric parameters (Å, °)

**Table:**

C6—C5	1.3791 (16)	C8—C9	1.4730 (16)
C6—C7	1.3903 (15)	C8—C10	1.5460 (16)
C6—C8	1.5193 (15)	C8—H8	0.9800
C7—C2	1.3843 (16)	C10—C11	1.4593 (16)
C7—H7	0.9300	C10—H10A	0.9700
C5—C4	1.386 (2)	C10—H10B	0.9700
C5—H5	0.9300	C9—N1	1.1365 (16)
C2—O1	1.3623 (15)	O1—C1	1.4205 (17)
C2—C3	1.3890 (17)	C1—H1A	0.9600
C3—C4	1.371 (2)	C1—H1B	0.9600
C3—H3	0.9300	C1—H1C	0.9600
C4—H4	0.9300	C11—N2	1.1368 (16)
			
C5—C6—C7	120.25 (11)	C6—C8—C10	113.36 (9)
C5—C6—C8	119.48 (10)	C9—C8—H8	108.3
C7—C6—C8	120.28 (9)	C6—C8—H8	108.3
C2—C7—C6	119.71 (10)	C10—C8—H8	108.3
C2—C7—H7	120.1	C11—C10—C8	111.38 (9)
C6—C7—H7	120.1	C11—C10—H10A	109.4
C6—C5—C4	119.46 (12)	C8—C10—H10A	109.4
C6—C5—H5	120.3	C11—C10—H10B	109.4
C4—C5—H5	120.3	C8—C10—H10B	109.4
O1—C2—C7	124.16 (11)	H10A—C10—H10B	108.0
O1—C2—C3	115.90 (11)	N1—C9—C8	179.20 (13)
C7—C2—C3	119.94 (11)	C2—O1—C1	118.40 (10)
C4—C3—C2	119.79 (11)	O1—C1—H1A	109.5
C4—C3—H3	120.1	O1—C1—H1B	109.5
C2—C3—H3	120.1	H1A—C1—H1B	109.5
C3—C4—C5	120.84 (12)	O1—C1—H1C	109.5
C3—C4—H4	119.6	H1A—C1—H1C	109.5
C5—C4—H4	119.6	H1B—C1—H1C	109.5
C9—C8—C6	110.45 (9)	N2—C11—C10	178.53 (13)
C9—C8—C10	108.02 (9)		
			
C5—C6—C7—C2	−0.64 (18)	C6—C5—C4—C3	0.3 (2)
C8—C6—C7—C2	179.32 (11)	C5—C6—C8—C9	118.72 (12)
C7—C6—C5—C4	0.4 (2)	C7—C6—C8—C9	−61.25 (14)
C8—C6—C5—C4	−179.55 (13)	C5—C6—C8—C10	−119.90 (12)
C6—C7—C2—O1	−179.11 (11)	C7—C6—C8—C10	60.14 (14)
C6—C7—C2—C3	0.13 (19)	C9—C8—C10—C11	−177.45 (10)
O1—C2—C3—C4	179.91 (14)	C6—C8—C10—C11	59.81 (13)
C7—C2—C3—C4	0.6 (2)	C7—C2—O1—C1	−9.4 (2)
C2—C3—C4—C5	−0.8 (2)	C3—C2—O1—C1	171.30 (14)

### Hydrogen-bond geometry (Å, °)

**Table:**

*D*—H···*A*	*D*—H	H···*A*	*D*···*A*	*D*—H···*A*
C8—H8···N1^i^	0.98	2.61	3.4864 (17)	150
C10—H10A···O1^ii^	0.97	2.38	3.2470 (15)	149
C10—H10B···N2^iii^	0.97	2.60	3.4823 (18)	151

Symmetry codes: (i) *x*+1, *y*, *z*; (ii) −*x*+1, *y*+1/2, −*z*+3/2; (iii) *x*−1, *y*, *z*.
